# Insecticide Resistance Status of *Aedes aegypti* Adults and Larvae in Nouakchott, Mauritania

**DOI:** 10.3390/insects16030288

**Published:** 2025-03-11

**Authors:** Mohamed Haidy Massa, Mohamed Aly Ould Lemrabott, Nicolas Gomez, Ali Ould Mohamed Salem Boukhary, Sébastien Briolant

**Affiliations:** 1 Unité de Recherche Génomes et Milieux (GEMI), Université de Nouakchott, Nouveau Campus Universitaire, Nouakchott BP 5026, Mauritania; medhaidy@gmail.com (M.H.M.); mohamedalylemrabott@yahoo.fr (M.A.O.L.); alimedsalem@gmail.com (A.O.M.S.B.); 2 Institut de Recherche Biomédicale des Armées (IRBA), Département Risques Vectoriels, Unité Parasitologie et Entomologie, 13005 Marseille, France; nico13dna@hotmail.com; 3 Unité Mixte de Recherche Risques Infectieux Tropicaux et Microorganismes Emergents, Assistance Publique-Hôpitaux de Marseille, Service de Santé des Armées, Aix Marseille University, 13005 Marseille, France; 4 Institut Hospitalo-Universitaire Méditerranée Infection, 13005 Marseille, France

**Keywords:** *Aedes aegypti*, arbovirus, *Bti*, insecticide resistance, *kdr*, larvicides, temephos, pyrethroids, Mauritania

## Abstract

*Aedes aegypti* mosquitoes are established throughout Nouakchott, Mauritania, where its insecticide resistance status is unknown and dengue has become endemo-epidemic since 2014. Eggs were collected using ovitraps at 12 sites in five districts of Nouakchott, in August 2024. Adult females and larvae of the F1 generation were used for bioassays. Permethrin, deltamethrin, bendiocarb, and malathion were evaluated at discriminating concentrations. Larval assays were carried out at seven concentrations with *Bacillus thuriengensis* var *israelensis* (*Bti*) and temephos. The presence of knockdown resistance (*kdr*) mutations known to be associated with pyrethroid resistance was assessed by polymerase chain reaction and amplicons sequencing. Adults showed high levels of resistance to all insecticides tested. Larvae were susceptible to *Bti* (LD50 < 50 µg/L) and temephos (LD50% = 6.8 ± 0.7 µg/L). Only three *kdr* point mutations, S989P, V1016G, and F1534C, were found. The tri-locus genotypes SP/VG/FC were significantly associated with pyrethroid survival while only the tri-locus genotypes PP/GG/FF showed significant association with deltamethrin resistance. Given their level of insecticide resistance, there is an urgent need to control *Ae. aegypti* populations by several methods, including the use of biological larvicides, physical elimination of peridomestic breeding sites, water drainage, and public education to prevent arbovirus transmission.

## Simple Summary

*Aedes aegypti* mosquitoes, key vectors of arboviruses, represent a serious threat to the public health of the inhabitants of Nouakchott, where recurrent dengue epidemics have occurred in the city since 2014. The insecticide resistance status of adult mosquitoes and larvae was not well known. Adults showed strong resistance to the four insecticides tested (permethrin, deltamethrin, bendiocarb, and malathion) belonging to three insecticide families. However, the larvae were susceptible to certain chemicals such as temephos, or to biological larvicides such as toxins from the bacterium *Bacillus thuriengensis* var *israelensis*. Three genetic mutations associated with pyrethroid resistance have been identified and should facilitate the monitoring of pyrethroid resistance in *Aedes aegypti* mosquitoes. Various control methods, such as the use of biological larvicides and the elimination of breeding sites, are therefore urgently needed to combat dengue transmission.

## 1. Introduction

Several arboviruses of medical importance circulate in Mauritania, including yellow fever virus (YFV), Rift Valley fever virus (RVFV), Crimean–Congo hemorrhagic fever virus (CCHFV), dengue virus (DENV) and chikungunya virus (CHIKV) [1]. Among these viruses, YFV, DENV, and CHIKV can be transmitted by *Aedes* mosquitoes [2]. The global incidence of dengue has increased 30-fold over the last 50 years [3], and it is estimated that between 100 and 400 million people are infected each year [4].

In Mauritania, the first dengue epidemic occurred in October–November 2014 in Nouakchott, [5,6]. Fourié et al. confirmed the circulation of DENV-2 during the 2014 epidemic in Nouakchott [5]. In October 2015, the presence of DENV-1 was also demonstrated in a French traveler returning from Mauritania by the Centre national de référence des arbovirus (Marseille, France) [7]. More recently, in 2018, in the town of Rosso near the border with Senegal, DENV-2 was also reported in a pool of RT PCR-positive *Aedes aegypti* mosquitoes [8]. In 1987, during the first outbreak of RVFV reported in Mauritania, 24 cases of yellow fever were diagnosed by ELISA, one of which was confirmed by virus isolation [9]. Since then, no cases of yellow fever have been reported in Mauritania. In a cross-sectional study of 1300 non-febrile patients attending the Nouakchott national hospital in 2021, a seroprevalence of 2.8% was found for CHIKV for the first time in Mauritania [10]. Finally, Zika virus (ZIKV) has recently been reported in Africa [11,12], notably in Senegal, where its circulation in mosquitoes [13] indicates a risk of introduction into Mauritania.

Entomological surveys have revealed the presence of *Ae. aegypti* in Nouakchott and its probable role in the transmission of DENV during the 2014 epidemic [6,14]. To date, there is no specific treatment or effective prophylaxis against most of the diseases transmitted by *Ae. aegypti*. Spatial spraying of insecticides remains a commonly used method for rapidly controlling mosquito populations during arbovirus epidemics [15]. However, most vector control strategies face operational challenges related to the emergence and development of insecticide resistance through various mechanisms. Insecticide resistance in *Ae. aegypti* and/or *Ae. albopictus* mosquitoes have been reported in several West African countries, including Senegal [16], Burkina Faso [17,18,19,20], Benin [21], Côte d’Ivoire [22], and Niger [23]. For the control of *Ae. aegypti* larvae, *Bacillus thuringiensis* var. *israelensis* (*Bti*) is one of the most effective larvicides, with proven efficacy in control programs around the world [24,25,26] and for which no resistance has yet been documented, unlike temephos, for which resistance has been demonstrated in several studies [27,28,29].

In Mauritania, there is no real vector control program apart from random campaigns of spatial spraying with insecticides. The ecology, behavior, and susceptibility of *Ae. aegypti* to insecticides are almost totally unknown, even though they are a prerequisite for the success of a suitable and effective vector control program. A single molecular study was carried out, in 2018–2019, on *Ae. aegypti* specimens from Nouakchott, showing the presence of several mutations in the voltage gate sodium channel (*vgsc*) gene, known to confer the knockdown resistance (*kdr*) mechanism to pyrethroids [30]. The limitation of that study was the absence of resistance phenotype to pyrethroids.

The main objectives of our study were to evaluate phenotypes to insecticides of the pyrethroid, carbamate, and organophosphate families, as well as to two larvicides, *Bti* and temephos, in the *Ae. aegypti* population of Nouakchott. The secondary objective was to determine whether there was an association between kdr genotypes and pyrethroid resistance phenotypes.

## 2. Materials and Methods

### 2.1. Sampling Sites

**Figure 1:**
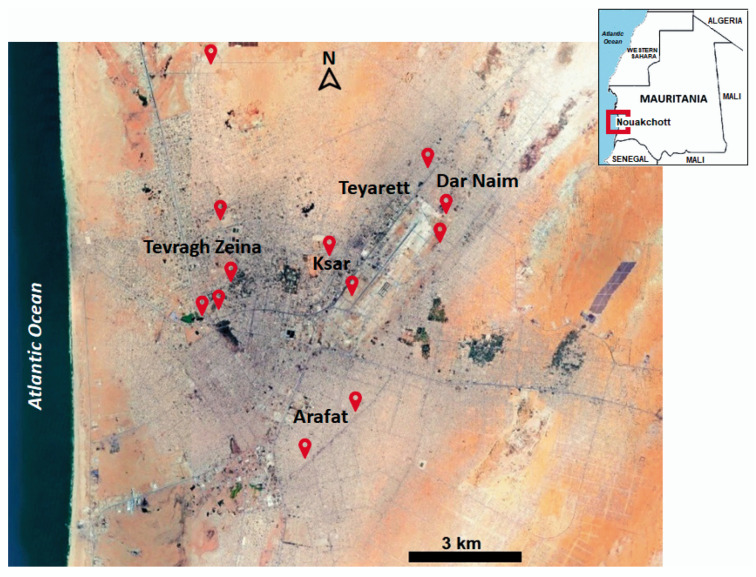
Location of ovitraps (in red) in the five districts of Nouakchott. Source: Google Maps (2025).

*Aedes aegypti* eggs were collected using ovitraps from 12 sites located in five out of nine districts of Nouakchott (Tevragh Zeina, Arafat, Ksar, Teyaret, Dar Naim), in August 2024, during the rainy season (Figure 1; Supplementary File S1). Nouakchott, the capital city of Mauritania, is situated in the Atlantic coastal zone; its climate is characterized as Saharan, i.e., low annual rainfall (<100 mm on average) and a mean annual temperature and humidity of 27 °C and 56.5%, respectively.

A total of 300 ovitraps were placed in the gardens or courtyards of houses at the 12 sites (2 or 3 ovitraps per house in around 10 houses around the GPS coordinates of each site), after obtaining oral informed consent from the owners of the gardens and houses. Each ovitrap consisted of a cylindrical black plastic container with a capacity of around 250 mL (7.0 cm diameter, 9.0 cm height) filled with tap water to a level corresponding to 2/3 of its height. Each ovitrap contained a wooden board, one side of which was porous to retain the eggs.

### 2.2. Mosquito Rearing

The eggs were then transported to the Institut de Recherche Biomédicale des Armées in Marseille and hatched in reverse osmosis water. Larvae from the various collection sites were then combined in equal proportions and reared on a standardized daily diet of fish feed in 24 × 34 × 9 cm plastic trays at a density of around 400 larvae per tray. A maximum of 500 adult males and females were maintained in 24 × 24 × 24 cm cages with permanent access to a 10% sucrose solution. Larvae and adults were maintained under standardized rearing conditions (28 °C, 75 ± 5% relative humidity, 12:12 h light–dark cycle). Adult females were fed twice weekly for 30 min with human blood obtained from the Etablissement Français du Sang (EFS) via a membrane feeding system (Hemotek Ltd., Blackburn, UK) using parafilm as membrane. Access to human blood was based on an agreement with EFS. All adult female mosquitoes of the F0 generation were identified as *Ae. aegypti* on the basis of a morphological identification key [31]. F1 generation eggs were collected daily and stored until immersion in water to do larvicidal and adulticidal bioassays.

### 2.3. WHO Bioassays

#### 2.3.1. Adulticidal Bioassays

Insecticide susceptibility tests for adult females *Ae. aegypti* followed WHO recommendations [32]. Insecticide-treated papers supplied by WHO (Vector Control Research Unit at the University of Science, Penang, Malaysia) were used at discriminant concentrations to assess the susceptibility profile of *Ae. aegypti* in Nouakchott to different classes of insecticides, namely type I and type II pyrethroids, permethrin (0.4%) and deltamethrin (0.03%), respectively, the organophosphate malathion (1.5%) and the carbamate bendiocarb (0.2%). For each dose of insecticide, approximately 25 non-blood-fed female mosquitoes older than 3 days of age were introduced into each tube with insecticide-impregnated paper. A total of 100 mosquitoes were exposed for 1 h to each insecticide and transferred to observation tubes, with access to a cotton swab soaked in 10% sucrose solution. Mortality was assessed after 24 h, alive and dead specimens were separated in cages, thrown for 30 min, and then individually placed in 96 well-plates at −80 °C before being used for molecular screening of resistance mechanisms. For each bioassay, in parallel, two control tubes were used for pyrethroids (with impregnated paper with acetone and silicon oil) or carbamate and organophosphate (with impregnated paper with acetone and olive oil).

#### 2.3.2. Larval Bioassays

In line with WHO recommendations [33], late third-instar or young fourth-instar larvae of the F1 generation were used, to determine their susceptibility to the two larvicides: *Bti* (500 g, VectoBac strain AM65-52, Edialux, Replonges, France) and temephos (250 mg; Sigma-Aldrich, St Louis, MO, USA). Seven different concentrations were tested in three independent experiments for each larvicide to determine the lethal dose (LD) required to kill 25% (LD25), 50% (LD50), and 95% (LD95) of the larvae. Temephos was used at 0, 3.75, 7.5, 15, 30, 60, 125 and 250 μg/L, and *Bti* at 0, 50, 100, 200, 400, 600, 800 and 1000 μg/L. Four replicates of 25 larvae were used at each concentration (for a total volume of 100 mL) in plastic cups. The control corresponded to larvae in 100 mL of water without insecticide for *Bti* and 99 mL of water plus 1 mL of absolute ethanol for temephos.

### 2.4. Genotyping kdr

After exposure to deltamethrin 0.03% and permethrin 0.4%, 64 and 75 specimens of live *Ae. aegypti* and 32 and 21 specimens of dead *Ae. aegypti*, respectively, were crushed in a TissueLyser II (Qiagen S.A.S., Courtaboeuf, France) and DNA was extracted as previously described [30].

Primer pairs vgsc V410L F (forward primer, 5′-ATTATCCCCACTCTCCCCCT-3′) and vgsc V410L R (reverse primer, 5′-TTGCACACATACACACACGG-3′), 0.625 μM each, were used to genotype V410L mutation. Genotyping of L982W, S989P, I1011V/M, A1007G, V1016G/I, T1520I, I1532T, and F1534C/S/L mutations have been already described in Ould Lemrabott et al. [30]. The same amplification conditions were applied to genotype V410L. The sequences were analyzed using Geneious Prime software version 2022.2.2.

### 2.5. Statistical Analyses

The proportions of dead or alive females after exposure to deltamethrin or permethrin according to the *kdr* genotype were compared using Fisher’s exact test. For the larvae, LD25, LD50, and LD95 were obtained by using BioRssay package [34]. Hardy–Weinberg equilibrium was calculated using the Chi-square test. Statistical significance was considered for *p* < 0.05. All data were analyzed using GraphPad Prism^®^ version 7.00 or R software v4.4.2 [35].

## 3. Results

### 3.1. Adult Bioassays

**Figure 2:**
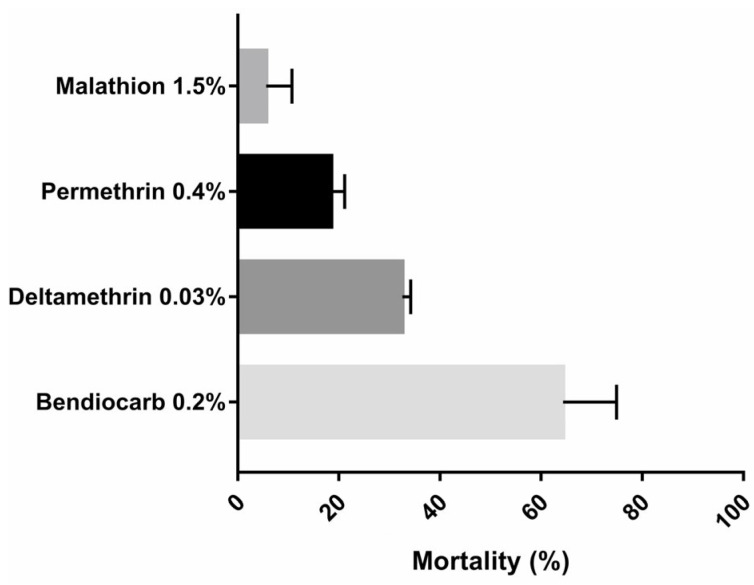
WHO tubes bioassay mortality of *Aedes aegypti* exposure to insecticides at discriminant concentrations. Error bars represent the standard deviation of the mean.

All negative controls showed 0% mortality after 24 h. For the F1 generation of the Nouakchott *Ae. aegypti* population, low mortality rates were observed with all insecticides tested (Figure 2). For the organophosphate insecticide, the adult mortalities were 5.6 ± 5.1% for 1.5% malathion. For the pyrethroids, the mortality rates were 18.5 ± 2.7% and 32.6 ± 1.6% for 0.4% permethrin and 0.03% deltamethrin, respectively. For the carbamates, adult mortalities were 64.3 ± 10.6% for 0.2% bendiocarb.

### 3.2. kdr Genotypes and Association with Pyrethroid Resistance

All successfully genotyped mosquitoes were wild-type homozygous (i.e., monomorphic) for *kdr* mutations 410 (*n* = 182), 982 (*n* = 93), 1007 (*n* = 180), 1011 (*n* = 180), 1520 (*n* = 189) and 1532 (*n* = 190) (Table 1).

The other three *kdr* point mutations, 989P, 1016G, and 1534C, were found with allelic frequencies of 0.29, 0.28, and 0.62, respectively. The most frequently detected genotypes were heterozygous 1534FC, 989SP, and homozygous 1016VV, with frequencies of 48%, 44%, and 45%, respectively. Homozygous wild-type 1534FF and homozygous mutant-type 989PP and 1016GG had the lowest frequencies (13%, 6%, and 5%, respectively). Genotype distribution did not deviate from Hardy–Weinberg equilibrium for any of the three mutations (S989P, V1016G, and F1534C).

**Figure 3:**
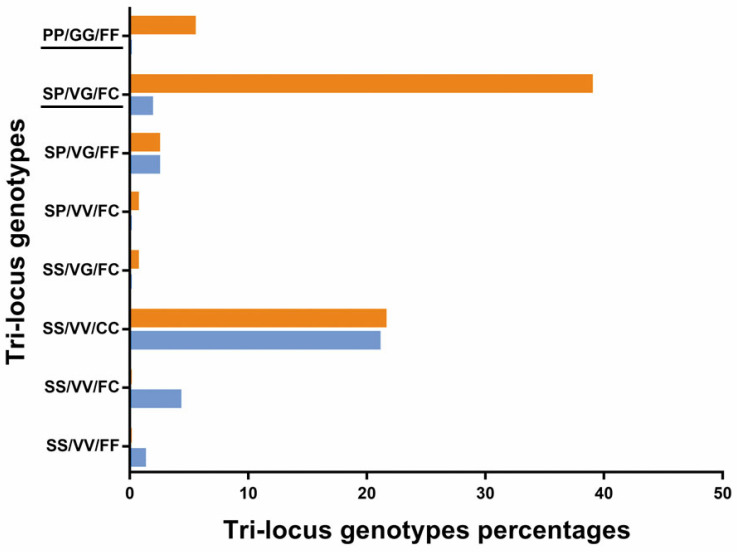
Percentages of tri-locus genotypes combination of S989P, V1016G, and F1534C *kdr* mutations in dead (blue) and alive (orange) pyrethroid-exposed *Aedes aegypti*. Genotypes underlined in black indicate genotypes associated with pyrethroid resistance.

Eight genotypes were found out of twenty-seven possible genotype combinations for the three *kdr* mutations S989P, V1016G, and F1534C on the basis of DNA sequencing (Figure 3).

The most common genotype was the homozygous 1534 SS/VV/CC mutant (42.5%), followed by the triple-heterozygous mutant for the three SP/VG/FC mutations (40.7%) and the triple homozygous wild-type SS/VV/FF (1.2%). Six haplotypes were observed: SVF (35.1%), SVC (31.5%), PGC (27.4%), PGF (5.2%), SGC (0.4%), and PVC (0.4%).

The tri-locus genotype SP/VG/FC was significantly associated with permethrin survival compared with the reference genotype SS/VV/FF and SS/VV/FC, for which there were no survivors (Table 2).

The tri-locus genotypes SP/VG/FC and PP/GG/FF were significantly associated with deltamethrin resistance compared with the same reference genotype as for permethrin (Table 3).

The tri-locus PP/GG/CC homozygous resistant genotype was not observed in any mosquitoes tested with permethrin and deltamethrin.

### 3.3. Larval Bioassays

The LD25, LD50 and LD95 for larvae were 4.5 ± 0.3 μg/L, 6.8 ± 0.7 µg/L and 18.7 ± 3.1 µg/L, respectively, for temephos (Supplementary File S2). No mortality was observed in any of the plastic control cups (for temephos and *Bti*). For *Bti*, LD25, LD50, and LD95 could not be determined due to the very high susceptibility of *Ae. aegypti* larvae. For *Bti*, the LD50 was less than 50 µg/L, corresponding to the lowest concentration tested.

## 4. Discussion

The present study is the first to assess the insecticide resistance status of adults and larvae of *Ae. aegypti* in Nouakchott, Mauritania, despite the fact that it is a vector of dengue fever, which is now endemo-epidemic there. Results from adult bioassays showed that *Ae. aegypti* is phenotypically highly resistant to permethrin (type I pyrethroids), deltamethrin (type II pyrethroids), bendiocarb (carbamate), and malathion (organophosphate), with mortality rates of 18.5% for 0.4% permethrin, 32.6% for 0.03% deltamethrin, 64.3% for 0.2% bendiocarb and 5.6% for 1.5% malathion, respectively. For *Ae aegypti* and *Ae. albopictus*, discriminant insecticide concentrations for adult bioassays were established by WHO in 2016 and updated in 2022 [32,33]. Nevertheless, in most studies assessing *Ae. aegypti* resistance phenotypes to insecticides, the discriminating concentrations of *Anopheles* are always used for pyrethroids and malathion, which are higher than those of *Ae. aegypti*, which could lead to an underestimation of susceptibility to these insecticides. In any case, in several studies carried out in West Africa, notably in certain localities in Senegal, Côte d’Ivoire, Ghana, and Nigeria [16,36,37,38,39], and in Central Africa such as Cameroon and Congo Brazzaville [40,41], *Ae. aegypti* mosquito populations show the same multi-resistant phenotype to pyrethroids, carbamates, and organophosphates as in Mauritania. However, in other West African countries, such as Burkina Faso and Niger [20,23], *Ae. aegypti* populations are resistant only to pyrethroids and remain susceptible to carbamates and organophosphates. In East Africa, notably in Ethiopia [42], *Ae. aegypti* populations are susceptible to all insecticides except carbamates.

In line with our previous report [30], only three *kdr* mutations associated with pyrethroid resistance were found in the present study in the *Ae. aegypti* population of Nouakchott with the following allelic frequencies: S989P (0.29), V1016G (0.28), and F1534C (0.62). Furthermore, all resistant mosquitoes (alive at the end of adult bioassays) were at least homozygous with a single mutation (SS/VV/CC) (42.3%) or double homozygous mutants (PP/GG/FF) (6%), or double heterozygous mutants (SP/VG/FF), (SP/VV/FC), (SS/VG/FC) (6%) or triple heterozygous mutants (SP/VG/FC) (40.5%). Although the mechanisms of metabolic, cuticular, or behavioral resistance need to be explored, it seems that resistance to pyrethroids in the *Ae. aegypti* population of Nouakchott is only molecular.

In several African countries (Angola, Burkina Faso, Cameroon, Côte d’Ivoire, and Niger), three *kdr* mutations are always associated with pyrethroid resistance: V410L, V1016I and F1534C [23,36,40,43,44]. The *kdr* mutation profile of *Ae. aegypti* mosquito populations in these countries appear to be identical to that observed in South America (Venezuela, Brazil, and Argentina), as demonstrated by several studies [45,46,47]. The *kdr* mutation profile of *Ae. aegypti* mosquito populations in Nouakchott (S989P, V1016G, and F1534C) is equivalent to that found in Asian countries [48]. The same *kdr* profile has also been detected in Indonesia [49], Saudi Arabia [50], and Benin [51], with the exception of Nigeria, where only S989P and F1534C were detected [39]. In view of this *kdr* mutation profile, it would be interesting to determine the origin of the *Ae. aegypti* mosquito population in Nouakchott using population genetic markers.

Our study showed a high susceptibility of *Ae. aegypti* larvae in Nouakchott to *Bti*-based larvicides. Like many studies, including those carried out in Central Africa, Cabo Verde, and Tanzania [52,53,54], resistance to *Bti* has never yet been proven. Finally, *Ae. aegypti* larvae in Nouakchott were also susceptible to temephos, while adults were highly resistant to 1.5% malathion in bioassays, even though both molecules are organophosphates. Other studies have already reported contradictory results: resistance of larvae to temephos and susceptibility of adults to malathion [53,55].

Vector resistance to chemical controls highlights the need for integrated management strategies that focus on mechanical methods and education to reduce breeding sites and dependence on chemicals [56].

The main limitations of the present study were (i) the absence of *Ae. aegypti* laboratory-susceptible strains to be used as the primary test control to ensure the quality of insecticide-treated papers and (ii) to make comparisons to determine and firmly establish susceptibility to *Bti* and temephos in tests with larvae and (iii) the different *Ae. aegypti* populations sampled having been grouped together, we have an average measure of the level of insecticide resistance. We can, therefore, conclude that, on average, the Nouakchott *Ae. aegypti* population is resistant to all the insecticide families tested. If we really want to answer the question of whether these results are the same in all the populations sampled, we will need to carry out a population genetic analysis to see whether or not there is a population structure of *Ae. aegypti* in the city.

## 5. Conclusions

*Aedes aegypti* mosquito populations in Nouakchott, Mauritania, are phenotypically resistant to carbamates, organophosphates, and pyrethroids. The latter resistance is probably entirely associated with three mutations S989P, V1016G, and F1534C in the voltage-gated sodium channel gene. In contrast, *Ae. aegypti* larvae are probably susceptible to *Bti* and temephos. As *Ae. aegypti* mosquitoes are established throughout Nouakchott and dengue has become endemo-epidemic since 2014, there is an urgent need to control *Ae. aegypti* populations. Given their level of resistance to different insecticide families, this objective can be achieved in several ways, including the potential use of biological larvicides or the physical elimination of peridomestic breeding sites, water drainage, public education, and the strengthening of entomological and arbovirus surveillance systems.

## Figures and Tables

**Table 1:** Allelic and genotypic frequencies of *kdr* mutations in *Aedes aegypti* mosquitoes, Nouakchott, Mauritania.

*kdr* Allele	N (%)				Frequency ^1^	χ2 HWE	*p*-Value
	SS	SR	RR	UI			
V410L	182 (95)	0 (0)	0 (0)	10 (5)	0	NA	NA
L982W	93 (48)	0 (0)	0 (0)	99 (52)	0	NA	NA
S989P	85 (44)	85 (44)	10 (6)	12 (6)	0.29	3.67	0.06
A1007G	180 (94)	0 (0)	0 (0)	12 (6)	0	NA	NA
I1011V	180 (94)	0 (0)	0 (0)	12 (6)	0	NA	NA
V1016G	86 (45)	79 (41)	10 (5)	17 (9)	0.28	2.22	0.14
T1520I	189 (98)	0 (0)	0 (0)	3 (2)	0	NA	NA
I1532T	190 (99)	0 (0)	0 (0)	2 (1)	0	NA	NA
F1534C	25 (13)	93 (48)	72 (38)	2 (1)	0.62	0.35	0.56

^1^ Frequency of mutant allele; S, susceptible; R, resistant; UI, uninterpretable; *kdr*, knockdown resistance; HWE, Hardy–Weinberg equilibrium at 5% significance level; NA, not applicable.

**Table 2:** Genotypes and their association with permethrin resistance in *Aedes aegypti* from Nouakchott, Mauritania.

Genotypes	Phenotypes		*p*-Value ^1^
	Dead (Susceptible)	Alive (Resistant)	
SS/VV/FF	1	0	Reference
SS/VV/FC	4	0	
SS/VV/CC	15	19	0.182
SS/VG/FC	0	1	0.333
SP/VV/FC	0	1	0.333
SP/VG/FF	2	3	0.524
**SP/VG/FC**	0	28	**<0.001**
PP/GG/FF	0	2	0.143

^1^ Fisher exact test; significantly associated genotypes are highlighted in bold.

**Table 3:** Genotypes and their association with deltamethrin resistance in *Aedes aegypti* from Nouakchott, Mauritania.

Genotypes	Phenotypes		*p*-Value ^1^
	Dead (Susceptible)	Alive (Resistant)	
SS/VV/FF	1	0	Reference
SS/VV/FC	3	0	
SS/VV/CC	20	17	0.128
SS/VG/FC	0	0	-
SP/VV/FC	0	0	-
SP/VG/FF	2	1	0.429
**SP/VG/FC**	3	37	**<0.001**
**PP/GG/FF**	1	7	**0.010**

^1^ Fisher exact test; significantly associated genotypes are highlighted in bold.

## Data Availability

Data are available from the corresponding author on reasonable request.

## Supplementary Materials

The following supporting information can be downloaded at: https://www.mdpi.com/article/10.3390/insects16030288/s1, Supplementary File S1: Geographic coordinates of ovitraps in the five districts of Nouakchott; Supplementary File S2: Mortality rate of *Aedes aegypti* larvae from Nouakchott exposed to temephos (mg/L).

## Abbreviations

The following abbreviations are used in this manuscript:

*Bti*	*Bacillus thuriengensis* var *israelensis*
CHIKV	chikungunya virus
CCHFV	Crimean–Congo haemorrhagic fever virus
DENV	dengue virus
EFS	Etablissement Français du Sang
ELISA	Enzyme Linked Immunosorbent Assay
*kdr*	knockdown resistance
LD	lethal dose
RVFV	Rift Valley fever virus
RT PCR	Real Time Polymerase Chain Reaction
WHO	World Health Organization
YFV	yellow fever virus
ZIKV	Zika virus
