# Supplementary material for: Insecticide Resistance Status of Aedes aegypti Adults and Larvae in Nouakchott, Mauritania
Source: Insects. 2025 Mar 11;16(3):288. doi: 10.3390/insects16030288 (PMC11942675; doi:10.3390/insects16030288)
Supplement: Supplementary file 1 [file insects-16-00288-s001.zip › Supplementary File S1.pdf]

Supplementary File S1. Geographic coordinates of ovitraps in the five districts of Nouakchott.

District	Site	Latitude North	Longitude West
Teyarett	1	18.129284	15.933954
Dar Naim	2	18.102521	15.929783
	3	18.101972	15.930179
Tevragh Zeina	4	18.086432	15.986423
	5	18.085152	15.991297
	6	18.098823	15.984658
	7	18.119833	15.994681
	8	18.160250	15.993972
Ksar	9	18.086919	15.933954
	10	18.103429	15.952836
Arafat	11	18.049951	15.965622
	12	18.054720	15.958407
