# Supplementary figures and images for: Insecticide Resistance Status of Aedes aegypti Adults and Larvae in Nouakchott, Mauritania
Source: Insects. 2025 Mar 11;16(3):288. doi: 10.3390/insects16030288 (PMC11942675; doi:10.3390/insects16030288)

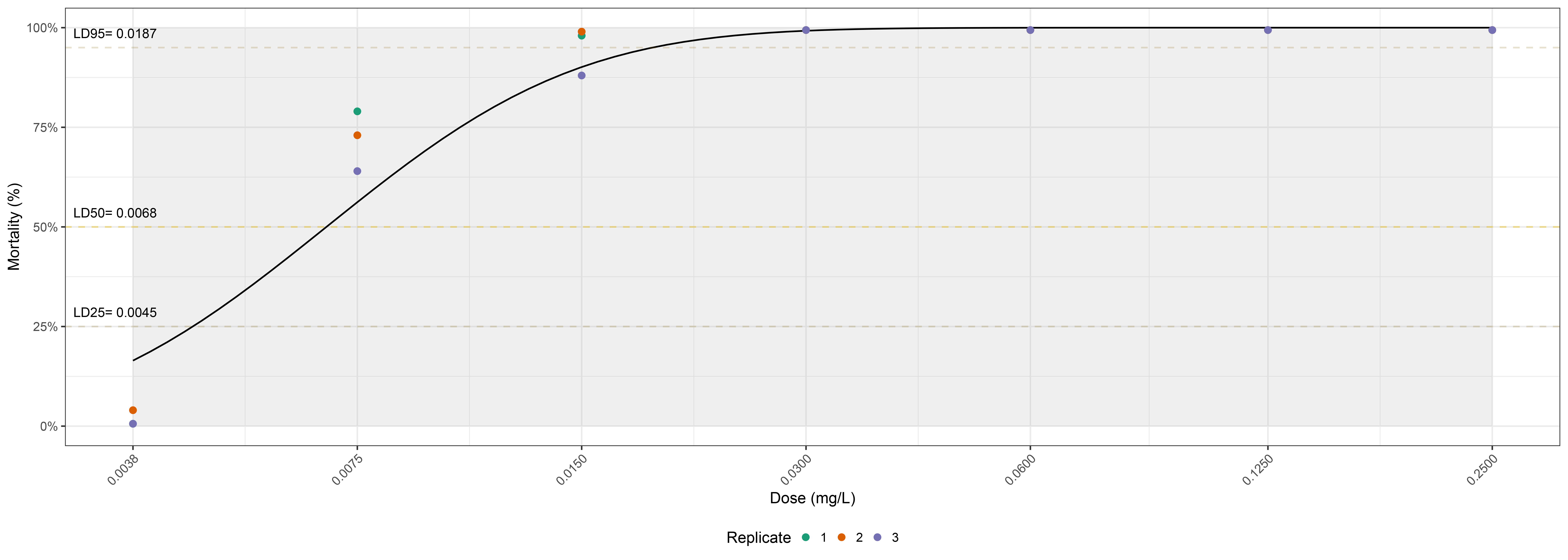

Supplement: Supplementary file 1 [file insects-16-00288-s001.zip › Supplementary File S2.TIFF]
